# Effect of Laser-Induced Optical Breakdown on the Structure of Bsa Molecules in Aqueous Solutions: An Optical Study

**DOI:** 10.3390/molecules27196752

**Published:** 2022-10-10

**Authors:** Egor I. Nagaev, Ilya V. Baimler, Alexey S. Baryshev, Maxim E. Astashev, Sergey V. Gudkov

**Affiliations:** Prokhorov General Physics Institute of the Russian Academy of Sciences, 119991 Moscow, Russia

**Keywords:** optical breakdown, laser radiation, acoustic oscillations, BSA, protein damage, optical properties of proteins, optical methods

## Abstract

The influence of laser radiation of a typical surgical laser on the physicochemical properties of the Bovine Serum Albumin (BSA) protein was studied. It was established that the physicochemical characteristics of optical breakdown weakly depend on the concentration of protein molecules. At the same time, the patterns observed for an aqueous solution of BSA irradiated with a laser for different time periods were extremely similar to the classical ones. It was established that after exposure to laser radiation, the optical density of protein solutions increases. At the same time, the intensity of BSA fluorescence due to aromatic amino acid residues decreases insignificantly after exposure to laser radiation. In this case, the position of the excitation and emission maximum does not change, and the shape of the fluorescence spot on 3D maps also does not change significantly. On the Raman spectrum after exposure to laser radiation, a significant decrease in 1570 cm^−1^ was observed, which indicates the degradation of *α*-helices and, as a result, partial denaturation of BSA molecules. Partial denaturation did not significantly change the total area of protein molecules, since the refractive index of solutions did not change significantly. However, in BSA solutions, after exposure to laser radiation, the viscosity increased, and the pseudoplasticity of aqueous solutions decreased. In this case, there was no massive damage to the polypeptide chain; on the contrary, when exposed to optical breakdown, intense aggregation was observed, while aggregates with a size of 400 nm or more appeared in the solution. Thus, under the action of optical breakdown induced by laser radiation in a BSA solution, the processes of partial denaturation and aggregation prevail, aromatic amino acid residues are damaged to a lesser extent, and fragmentation of protein molecules is not observed.

## 1. Introduction

Laser medical technologies are distinguished by their versatility, complexity, and diversity [1]. Laser medicine includes the effect of laser radiation on various parts of the body: skin, bones, muscles, adipose tissue, tendons, internal organs, eyes, lips, etc. [2]. All these parts of the body have a complex structure and their own properties, both optical (spectral characteristics, reflection coefficient, radiation penetration depth) and thermophysical (thermal conductivity, thermal diffusivity, heat capacity) [3]. In this regard, for each task, it is necessary to choose individual parameters of the laser irradiation regime: wavelength, exposure duration, power, pulse repetition rate, etc. [4]. To remove biological tissues, various scenarios for the interaction of laser radiation with an object are used: ablation (direct removal of a substance); coagulation; welding (connection); and crushing (using a shock wave) [5]. Overall, the laser appears to be an exceptionally accurate, versatile and user-friendly tool and has a great potential for medical applications [6]. In laser surgery, tissue destruction occurs directly in the process of exposure [7]. Currently, there are many indications for the use of lasers in surgery [8]. These are microsurgical operations (in particular on the eye), removal of small tumors, operations that require selective exposure (pigment spots and other subcutaneous formations), recanalization of vessels, passages; the stopping of bleeding and operations on blood-saturated organs; and fabric welding. In laser surgery powerful lasers are used, and the radiation power density is sufficient for the removal, destruction, or thermal necrosis of cells, tissues, or other objects to be eliminated [9,10,11].

Today, the most common surgical lasers are Nd:YAG lasers [12]. This is due to the fact that most tissues at the wavelength of the Nd:YAG laser (1064 nm) have a low absorption coefficient [13]. The effective penetration depth of such radiation into tissues can be several millimeters and provides good hemostasis and coagulation [14]. Nd:YAG laser radiation can be delivered to the treatment area using fiber optic light guides, which is also an important competitive advantage, since it allows laser radiation to be delivered to the lower and upper gastrointestinal tract in a practically non-invasive way [15]. The development of nonlinear processes in tissue upon absorption of laser radiation underlies the medical action of a laser. One of the most frequently recorded nonlinear processes is optical breakdown, a fast, irreversible process of transformation of a medium from transparent to strongly absorbing, under the action of intense radiation [16,17]. Optical breakdown occurs when certain threshold values of the laser radiation energy density are exceeded [18]. At energies close to the threshold values, the development of optical breakdown has a probabilistic character [19]. It is known that optical breakdown occurs less frequently when the absorption of laser radiation is lower [20]. Studies of laser breakdown in liquids show that the presence of nanosized impurities leads to an increase in breakdown probability and a decrease in the threshold values of laser radiation energies [21]. It has been established that the process of optical breakdown of a liquid is much (several orders of magnitude) more intense in the presence of nanosized objects in the medium [22]. Protein molecules, due to their size, are also nanoscale objects. At the present time, it is not known whether protein molecules can lead to an increase in the probability of breakdown in aqueous solutions. In addition, little is known about the changes that occur in protein preparations after exposure to optical breakdown. To answer the above two questions, we used a standard Nd:YAG laser with a pulse duration of approximately 10 ns. Bovine Serum Albumin (BSA) was used as a model protein, as it is one of the most studied proteins, quite frequently used in biological studies [23]. Thus, in this work, for the first time, using the example of BSA protein, it was discovered how protein molecules affect the characteristics of optical breakdown in an aqueous medium, how the optical properties of protein molecules change after optical breakdown, and what changes occur in the structure of molecules.

## 2. Results

**Figure 1:**
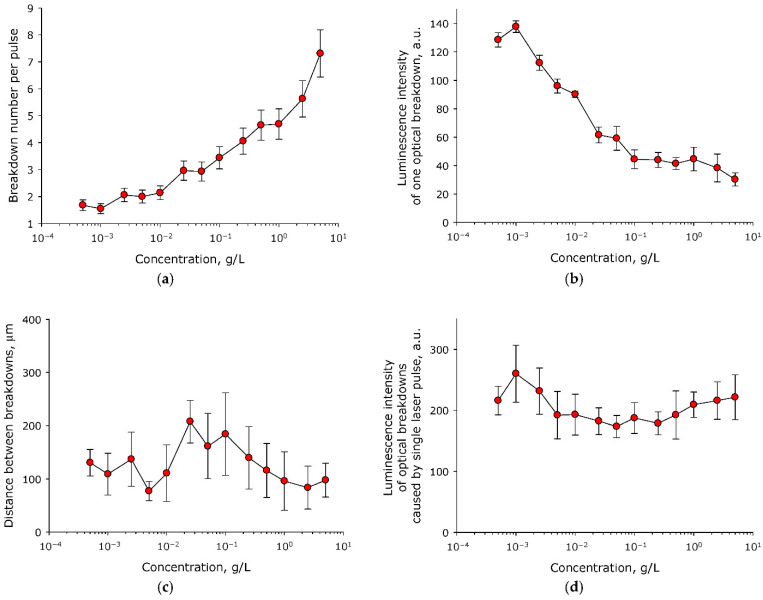
Effect of the concentration of BSA molecules in aqueous solution on the characteristics of optical breakdown plasma (*n* = 3, Mean ± SEM). (**a**) Effect of the concentration of BSA molecules in an aqueous solution on the number of optical breakdowns caused by a single laser pulse. (**b**) Effect of the concentration of BSA molecules in aqueous solution on the average luminescence intensity of individual optical breakdown. (**c**) Effect of the concentration of BSA molecules in an aqueous solution on the average distance between optical breakdowns caused by a single laser pulse. (**d**) Effect of the concentration of BSA molecules in an aqueous solution on the average luminescence intensity of optical breakdowns induced by a single laser pulse.

It is known that a single laser pulse in a transparent medium, depending on the energy density, can cause from one to several hundred micrometer-sized optical breakdowns. The number of optical breakdowns caused by a single laser pulse also depends on the number of nanosized seeds in the medium. Protein molecules can be considered such seeds, due to their nanometer size. The influence of the concentration of BSA molecules in aqueous solution on the characteristics of the optical breakdown plasma was studied (Figure 1). It was established that change in the concentration of BSA molecules in aqueous solution can have a significant effect on the number of optical breakdowns caused by a single laser pulse (Figure 1a). At low concentrations of BSA molecules (<0.01 g/L), approximately two optical breakdowns were observed per single laser pulse. As the concentration of BSA molecules increased, the number of optical breakdowns increased exponentially. Dependence basically can be described with the equation y = 2.2 + 4.2(1 − e^−1.6x^) (R^2^ = 0.89).

Usually, with an increase in the number of individual optical breakdowns induced by a single laser pulse, a decrease in the intensity of plasma formation in each individual optical breakdown is observed. The intensity of plasma formation was estimated from the luminescence intensity of individual optical breakdowns. It was shown that the concentration of BSA molecules in the aqueous solution had a significant effect on the average luminescence intensity of individual optical breakdown (Figure 1b). With an increase in the BSA concentration from 5 × 10^–4^ g/L to 0.1 g/L, the average intensity of the luminescence of individual optical breakdown decreased exponentially by almost three times, from 140 to 50 units. At concentrations of more than 0.1 g/L, the average intensity of the luminescence of individual optical breakdown did not change, and was in the range of 35–45 units.

It is known that in media containing nanosized objects, the distance between optical breakdowns induced by a single laser pulse is related to the concentration of these objects. Changing the average distance between laser pulses affects the hydrodynamics observed in solution. A change in hydrodynamic parameters can affect the scenario of the interaction of nanosized objects, both with each other and with laser radiation. The influence of the concentration of BSA molecules in aqueous solution on the average distance between optical breakdowns caused by a single laser pulse was studied (Figure 1c). It was shown that the distance between individual optical breakdowns of the medium, caused by a single laser pulse at all studied concentrations of BSA molecules lies in the range of 80–210 μm. Statistical differences are observed only between two experimental points (between concentrations of 5 × 10^−3^ and 5 × 10^−2^ g/L).

An important parameter of the efficiency of plasma formation and energy input during optical breakdown is the luminescence induced in the medium by a single laser pulse (the sum of the luminescence of all breakdowns induced by a single laser pulse). In fact, this parameter is an integral characteristic of the interaction efficiency of laser radiation with the medium. The effect of the concentration of BSA molecules in aqueous solution on the average luminescence intensity of optical breakdowns induced by a single laser pulse was studied (Figure 1d). It was shown that the average luminescence intensity of optical breakdowns induced by a single laser pulse did not change significantly over the entire range of concentrations studied.

**Figure 2:**
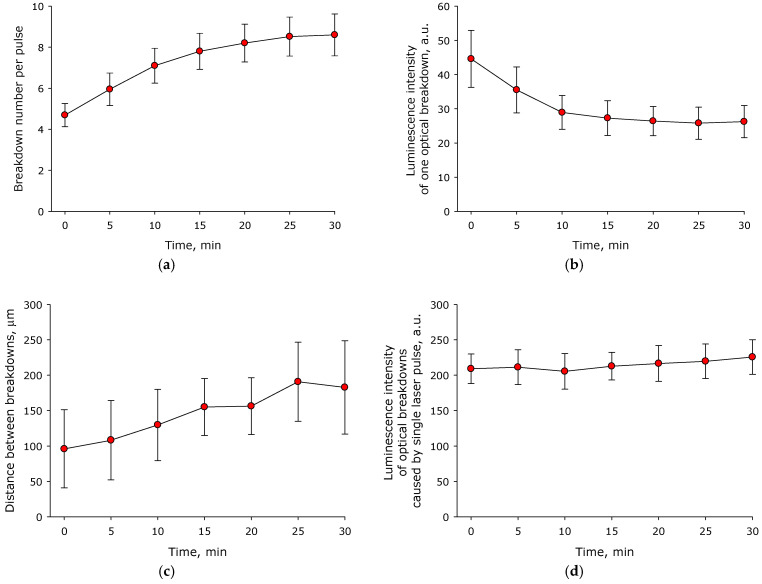
Influence of laser irradiation time of aqueous solution of BSA (1 mg/mL) on the characteristics of optical breakdown plasma (*n* = 3, Mean ± SEM). (**a**) Influence of the time of irradiation of an aqueous solution of BSA with laser on the number of optical breakdowns caused by a single laser pulse. (**b**) Effect of laser irradiation time of aqueous BSA solution on the average luminescence intensity of individual optical breakdown. (**c**) Influence of the time of irradiation of aqueous solution of BSA with laser on the average distance between optical breakdowns caused by a single laser pulse. (**d**) Effect of laser irradiation time of aqueous BSA solution on the average luminescence intensity of optical breakdowns induced by a single laser pulse.

It is known that during optical breakdown there is a change of the medium in which optical breakdown occurs. As a consequence, the physicochemical characteristics of optical breakdown depend on the time of exposure of the medium to laser radiation. The influence of the time of exposure to laser radiation, which causes optical breakdown of aqueous solution of BSA, on the number of optical breakdowns induced by a single laser pulse was studied (Figure 2a). It is shown that the number of optical breakdowns induced by a single laser pulse in a BSA solution increases from 5 to 9 after 30 minutes of exposure. The process can be described with the equation y = 0.1x + 5.3 (R^2^ = 0.89), where y—number of optical breakdowns caused by a single laser pulse, x—time in minutes. The influence of the time of irradiation of aqueous solution of BSA with laser on the average luminescence intensity of individual optical breakdown was estimated (Figure 2b). It is shown that after half an hour of laser irradiation, the average luminescence intensity of individual optical breakdown decreases by more than 40%. The process can be described with the equation y=19.3 + 0.1e^−0.1x^ (R^2^ = 0.99), where y—average luminescence intensity of individual optical breakdown, x—time in minutes. 

An estimate was made of the average distance between optical breakdowns caused by one laser pulse as a function of the time of exposure to laser radiation on an aqueous solution of BSA (Figure 2c). It was established that the average distance between individual breakdowns caused by a single laser pulse increased from 100 µm at the beginning of irradiation to 180 µm after 30 min of exposure to laser radiation. The influence of the time of irradiation of aqueous solution of BSA with a laser on the average luminescence intensity of all optical breakdowns induced by a single laser pulse was estimated (Figure 2d). It was shown that the average intensity of the luminescence of optical breakdowns caused by a single laser pulse did not change during at least 30 min of exposure to laser radiation on aqueous solutions of BSA. In this case, during the entire 30 min of exposure to laser radiation, there was a trend towards an increase in the average luminescence intensity (the intensity increased by 10% in 30 min).

**Figure 3:**
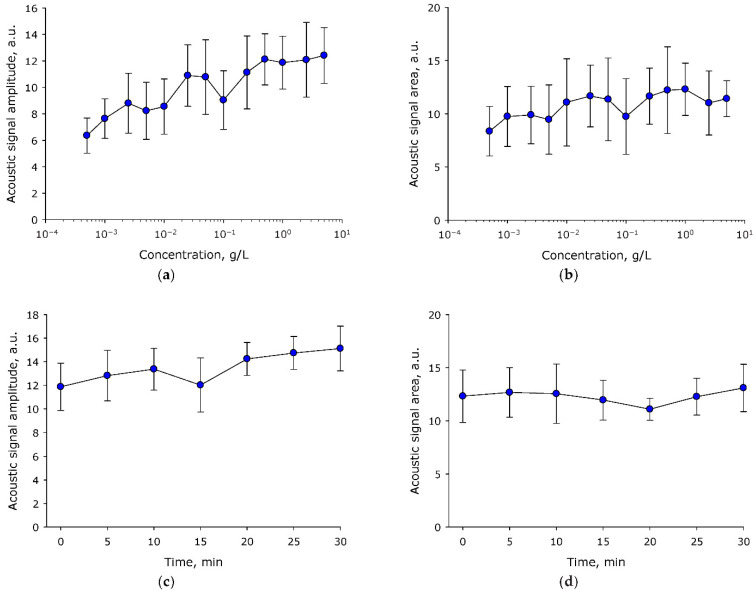
Influence of BSA aqueous solution irradiation time and its concentration on the characteristics of acoustic oscillations induced by optical breakdown (*n* = 3, Mean ± SEM). (**a**) Effect of the concentration of BSA molecules in aqueous solution on the average amplitude of acoustic vibrations induced by optical breakdown. (**b**) Effect of the concentration of BSA molecules in aqueous solution on the average intensity of acoustic vibrations induced by optical breakdown. (**c**) Effect of the time of irradiation of aqueous solution of BSA (1 mg/mL) with a laser on the average amplitude of acoustic oscillations induced by optical breakdown. (**d**) Influence of the time of irradiation of aqueous solution of BSA (1 mg/mL) with a laser on the average intensity of acoustic oscillations induced by optical breakdown.

During optical breakdown in the medium, acoustic oscillations appear. They are caused by the cavitation and collapse of the plasma bunch. The amplitude and average intensity of acoustic oscillations are often used for an integral estimation of the efficiency of optical breakdown in the medium. The effect of the concentration of BSA molecules in aqueous solution on the average amplitude of acoustic oscillations induced by a single laser pulse was studied (Figure 3a). It was shown that the average amplitude of acoustic oscillations induced by optical breakdown increased with increasing BSA concentration. In general, the process can be described with the equation y = 7.1 + 4.2(1 − e^−^^58.8x^), where y = amplitude of acoustic vibrations, x = concentration of BSA molecules. The influence of the concentration of BSA molecules in aqueous solution on the average intensity of acoustic vibrations induced by optical breakdown was estimated (Figure 3b). It was established that the average intensity of acoustic vibrations induced by optical breakdown did not change significantly at all studied BSA concentrations. The influence of the time of irradiation of aqueous solution of BSA with a laser on the average amplitude and average intensity of acoustic oscillations induced by optical breakdown was studied (Figure 3c,d). It was shown that the average amplitude and average intensity of acoustic signals induced by optical breakdown did not change significantly during at least 30 min of exposure. At the same time, the amplitude of acoustic oscillations tended to increase with the increase of exposure time (increases in amplitude of 20% were observed within 30 min).

Thus, the data obtained in the study of plasma parameters and acoustic oscillations allows us to state that the efficiency of optical breakdown at protein concentrations in the range of 0.1–10 g/L is more uniformly distributed than in other studied concentrations. From this, it follows that this range of protein concentrations is the most suitable for further studies. Regarding the time factor, the physical parameters of optical breakdown change monotonically with time, which makes it possible to carry out the necessary interpolations.

**Figure 4:**
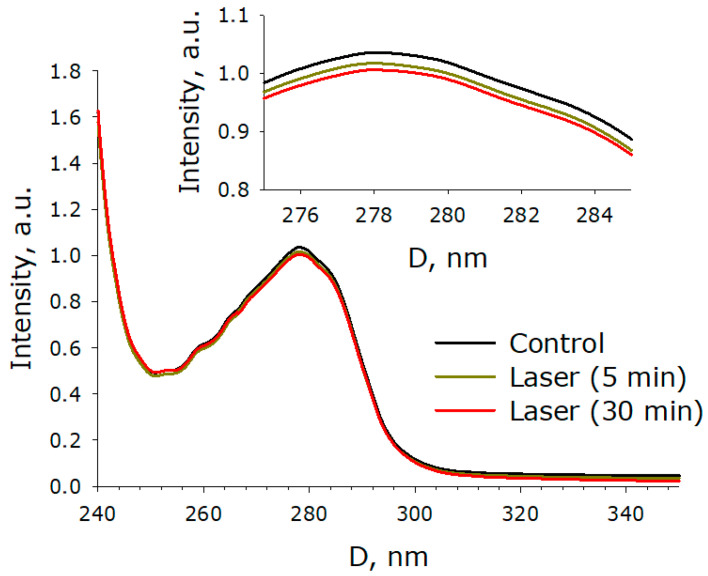
Effect of laser irradiation time on the optical density of an aqueous solution of BSA (*n* = 6–8, mean). The upper inset presents an enlarged view of the change in optical density in the region of the local maximum at 280 nm. The data were obtained using sub-nanometer differential two-beam spectroscopy.

The effect of laser irradiation time on the optical density of aqueous solution of BSA is shown in Figure 4. It is shown that under the action of laser radiation, the optical absorption of the BSA solution decreases. The absorption of aqueous solution of BSA decreases both in the local maximum (280 nm) and in the longer wavelength region (right shoulder of the peak, 310–350 nm). Thus, a decrease in the absorption intensity is observed both in the spectral absorption range of aromatic amino acid residues and in the range after 310 nm. Several scenarios for the development of events can be assumed. The first option is the degradation of aromatic amino acid residues. The second is due to partial denaturation or aggregation of the protein, or to non-specific scattering. 

**Figure 5:**
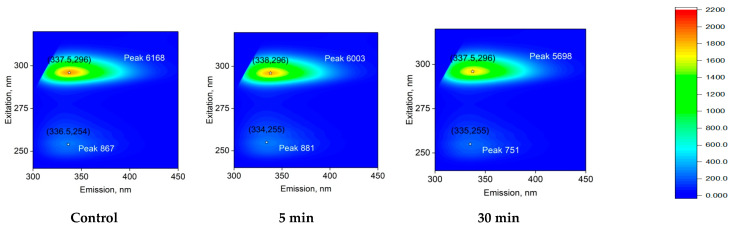
Effect of laser irradiation time on the fluorescence of BSA (5 mg/mL) protein solution (typical spectrum). 3D fluorescence maps are presented. The abscissa shows the range of emission wavelengths in nm (λ_em_). The ordinate shows the range of excitation wavelengths in nm (λ_ex_). The fluorescence intensity is expressed in relative units using a color scale, which is the same for all three spectra. The numbers in parentheses on the graphs indicate the local fluorescence maxima in the coordinates (λ_ex_; λ_em_). Fluorescence intensity is indicated with the word Peak and a number.

Figure 5 shows the effect of laser exposure time on the fluorescence of a BSA protein solution. It is shown that the fluorescence excitation maximum is observed at 296 nm. The position of the excitation maximum does not change after exposure of the BSA solution to laser radiation, both for 5 min and for half an hour. The fluorescence intensity decreases by 2% after 5 min of exposure to laser radiation, and by about 8% under the action of laser radiation for 30 min. The *maximum* intensity of emission for an intact protein solution and for a solution exposed to laser radiation is in the region of 337–338 nm. The shape of the fluorescence spot on 3D maps does not change significantly when exposed to laser radiation. The 3D fluorescence maps also show an order of magnitude lower intensity peak (λ_em_/λ_ex_ = 335/254 nm). When exposed to laser radiation for 30 min, a decrease in the intensity of this peak by 15% is observed. Thus, it was shown that under the action of laser radiation on protein solutions, a slight decrease in the fluorescence intensity of aromatic amino acid residues is observed. This change can be associated both with the degradation of aromatic amino acids and with a change in the secondary structure of the molecule. Raman spectroscopy was used in order to study the structure of the molecule.

**Figure 6:**
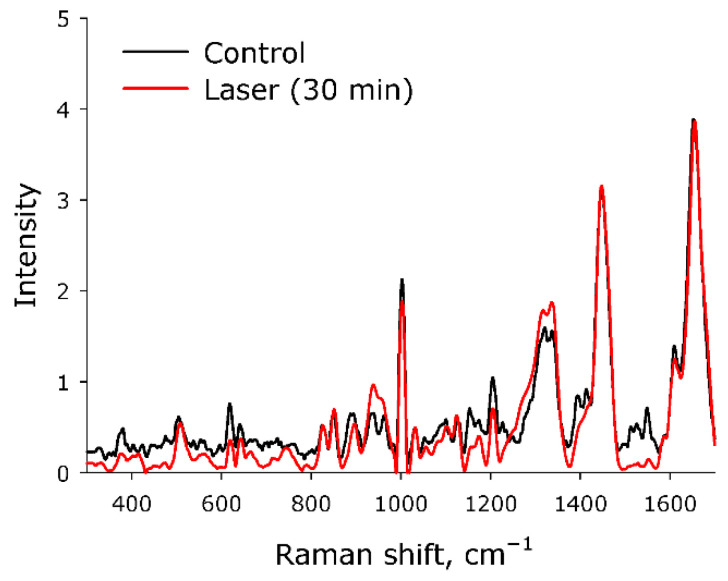
Raman spectra of BSA solutions for control and after 30 min laser exposure. The data were obtained with a Raman microscope. The intensity along the y-axis is presented in relative units.

Raman microscopy was used to study possible changes in the secondary structure of a protein molecule during optical breakdown. The Raman spectra of the native protein and the protein after exposure to laser radiation for 30 min were studied (Figure 6). It was found that after 30 min of exposure to laser radiation, no significant changes were observed in the protein spectra. However, the degradation of 1570 cm^−^^1^ should be noted. It is obvious that such a change in the intensity in the Raman signal can only indicate a slight rearrangement within the protein molecule. Thus, we know that structural changes have taken place, although we cannot unambiguously say what they are connected with. To clarify this, the refractive index of the BSA solution was measured and rheological studies were carried out.

**Figure 7:**
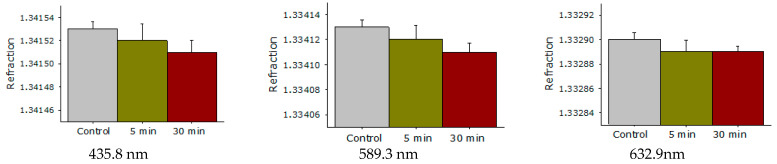
Effect of laser irradiation time on the refractive index of aqueous solution of BSA at wavelengths of 435.8 nm, 589.3 nm, 632.8 nm. Data obtained using precision refractometry (*n* = 3, Mean ± SD).

The effect of laser irradiation time on the refractive index of the BSA solution at wavelengths of 435.8 nm, 589.3 nm, and 632.8 nm was studied (Figure 7). It was shown that the BSA refractive index does not change significantly after exposure to laser radiation at all investigated wavelengths.

**Figure 8:**
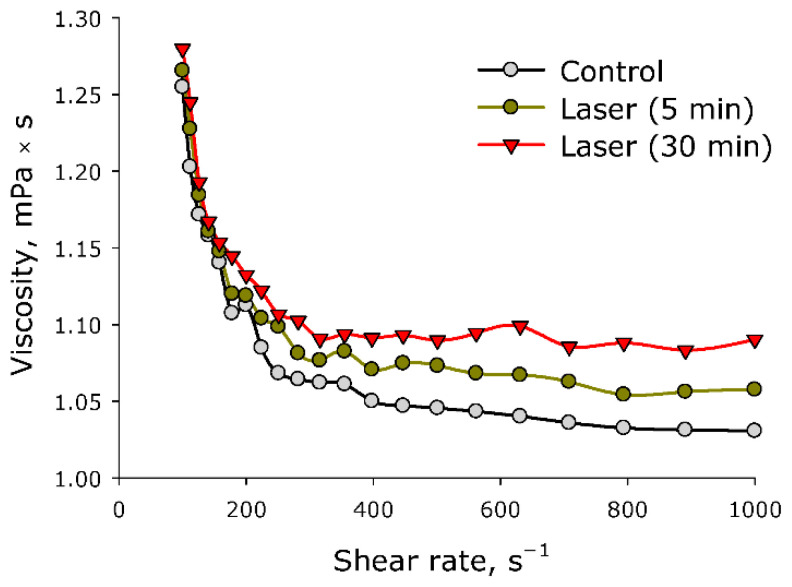
Effect of laser irradiation time on the viscosity of an aqueous solution of BSA at different shear rates (*n* = 3, Mean).

The effect of laser irradiation time on the viscosity of aqueous solution of BSA was studied at various shear rates (Figure 8). It was shown that aqueous solution of BSA molecules is characterized by pseudoplasticity. Pseudoplasticity is a property of fluid, characterized by the fact that the viscosity of the fluid decreases with increasing shear rate. The impact of laser radiation on aqueous solutions of BSA leads to an increase in viscosity. Moreover, at high shear rates, the viscosity of the control solution and the solution irradiated for 30 min differ by less than 10%. At low mixing rates, the viscosity of the control solution and the solution irradiated for 30 min does not differ. That is, laser irradiation led to a decrease in the pseudoplasticity of the BSA solution. Since at high shear rates (>400 s^−^^1^) the viscosity increases, the resistance to the movement of one part of the liquid to the other increases. In other words, protein molecules interact more intensively with each other, usually in protein solutions, and this is called aggregation. In order to test this assumption, the size evolution of light-scattering particles in an aqueous solution of BSA was studied.

**Figure 9:**
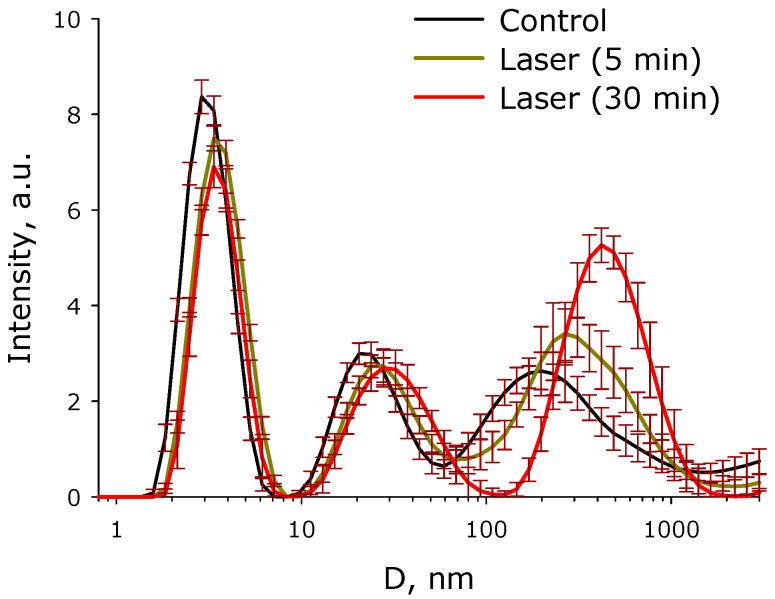
Effect of laser irradiation time on the evolution of the size distribution of BSA and its aggregates in aqueous solution (*n* = 5, Mean). The data were obtained by the dynamic light scattering (DLS) method.

The influence of the time of exposure to laser irradiation on the evolution of the BSA and its aggregates size distribution in an aqueous solution was studied (Figure 9). It was shown that the intact preparation contains individual BSA molecules (the average hydrodynamic diameter is approximately 3 nm), as well as aggregates (the average hydrodynamic diameters are 25 and 200 nm). There are ~1.1 × 10^6^ individual BSA molecules per one 25 nm aggregate in solution, and ~2.9 × 10^10^ individual BSA molecules per one 200 nm aggregate. When exposed to laser radiation, there is no increase in the average hydrodynamic diameter of individual BSA molecules. At the time of exposure to laser radiation, a decrease in the intensity of light scattering by 10–15% on individual molecules is clearly visible. For 30 min of laser exposure, the hydrodynamic diameter of “small” aggregates increases from 25 nm to 30 nm, and the hydrodynamic diameter of “large” aggregates increases from 200 nm to 400 nm. At the same time, the intensity of light scattering on such “large” aggregates increases by almost two times. After exposure to laser radiation for 30 min, one aggregate with a size of 400 nm accounted for ~3.7 × 10^12^ individual BSA molecules. The same number of “large” aggregates increased by more than 30 times. In general, the dose-dependent nature of the changes was visible. As the time of laser exposure increased, the changes in the evolution of the size distribution became more and more pronounced.

## 3. Discussion

Nanosized objects are usually considered as seeds for optical breakdown. It is known that the presence of nanosized objects in the medium significantly increases the probability of optical breakdown. In the literature, the influence of metal nanoparticles on the probability of optical breakdown and the main physicochemical processes occurring during optical breakdown are usually studied [24,25,26,27]. It has been established that the course of physicochemical processes occurring during optical breakdown of colloidal solutions of nanoparticles and protein molecules is different. For example, when the protein concentration changes by five orders of magnitude, the number of optical breakdowns caused by a single laser pulse increases by fewer than four times (Figure 1a). For comparison, when the concentration of metal nanoparticles (Au and Ni) changes by one order of magnitude, the number of optical breakdowns caused by a single laser pulse increases by a factor of three (Au) [28] or seven (Ni) times [29]. A change in the protein concentration in aqueous solution by five orders of magnitude changes the average luminescence intensity of individual optical breakdown by fewer than two times (Figure 1b). In the case of metallic nanoparticles, a change in the average luminescence intensity of individual optical breakdown by less than a factor of two is usually observed when the concentration changes by one order of magnitude [29]. The average distance between optical breakdowns caused by a single laser pulse has little dependance on the protein concentration (Figure 1c). The average distance between individual breakdowns induced by a single laser pulse in a protein solution is much smaller than in a colloidal solution of nanoparticles when exposed to a laser with very similar characteristics [28]. The average luminescence intensity of optical breakdowns caused by a single laser pulse also has its own characteristics. At high concentrations of nanoparticles, when the solution begins to become opalescent, the average luminescence intensity of optical breakdowns and other breakdown characteristics usually begin to decrease sharply [30], something not observed in the case of BSA protein molecules (Figure 1d). In this study, in addition to the effect of the concentration of protein molecules, we also studied the effect of the time of irradiation of an aqueous solution of BSA with a laser on the characteristics of the optical breakdown plasma (Figure 2). The regularities observed when aqueous solution of BSA was irradiated with a laser for different time periods were very similar to the regularities observed in aqueous colloidal solutions of metal nanoparticles [31].

The effect of BSA concentration on the characteristics of acoustic vibrations induced by optical breakdown was studied (Figure 3). It was shown that the average amplitude of acoustic oscillations induced by optical breakdown increases monotonically at all studied protein concentrations. In the case of optical breakdown of aqueous medium, pronounced “concentration” maxima (one or several) are always observed on nanoparticles, which significantly differ in intensity from the “basic” state [29]. At the same time, both during optical breakdown on individual nanoparticles and during optical breakdown on protein molecules, the average indicators of acoustic vibrations do not differ significantly [32].

A comprehensive analysis of BSA protein molecules was carried out using optical methods and viscometry. At the initial stage, it was found that after exposure to laser radiation, an increase in the intensity of absorption of protein solutions is observed (Figure 4). Moreover, an increase in optical density is observed both in the absorption range of aromatic amino acid residues and in the longer wavelength region (310 nm). It can be assumed that the data obtained may indicate changes in the protein structure [33]. It is known that optical breakdown produces a large number of both reducing and oxidizing equivalents [34]. The generation of ultraviolet radiation, shock acoustic waves, and microvolumes with a significant increase in temperature is observed [35]. With such a set of influences, the following can be observed: (1) chemical modification of amino acid residues; (2) fragmentation of the polypeptide chain; (3) change in the tertiary and secondary structure of BSA molecules; (4) partial denaturation; and (5) aggregation of molecules [36,37,38,39]. It should be noted that a similar set of events occurs with proteins under the action of oxidative stress [40], which develops in living systems under the action of various physical and chemical factors [41], during the development of inflammation [42], hypoxia [43], and a number of diseases [44], etc.

The fluorescence of protein solution of BSA after exposure to laser radiation was studied (Figure 5). Usually, the excitation maximum occurs in the wavelength range of 275–290 nm [45], and a maximum at 295 nm was recorded by the research team. This phenomenon is usually observed at high protein concentrations. Under the action of laser radiation, the fluorescence intensity somewhat decreases. In this case, no change in the emission maximum was observed. The chemical modification of the fluorophore usually affects the shape of the emission spot on the 3D fluorescence map. However, it was found that the shape of the fluorescence spot on 3D maps does not change significantly. Thus, it can be argued that under the action of optical breakdown, insignificant degradation of aromatic amino acid residues occurs. Raman microscopy was used to study possible changes in the secondary structure of the protein molecule during optical breakdown (Figure 6). No significant changes were observed in the BSA spectra, and the only change was observed in the region of 1570 cm−1. This change is associated with the degradation of α-helices [46]. Changes in the structure of α-helices usually lead to the partial denaturation or aggregation of molecules. With partial denaturation, there is a significant increase in the number of water molecules in the hydration shell of the protein, while a change in the refractive index is observed [47]. The refractive index was measured at different wavelengths with high accuracy (Figure 7) and no significant differences were observed. The aggregation of protein molecules, including “soft” ones, always leads to a change in rheological properties [48]. It was found that optical breakdown leads to an increase in the viscosity of aqueous BSA solutions, although it reduces the pseudoplasticity of the colloidal solution (Figure 8). Such changes in the solution may indicate a more intensive interaction of protein molecules with each other (aggregation) and the absence of fragmentation of the polypeptide chain. The study of the size evolution of light-scattering particles in an aqueous solution of BSA confirms the development of aggregation (Figure 9). Interestingly, after exposure to laser radiation, no shift of the peak of individual molecules to the region of smaller sizes was observed. Obviously, this indicates the absence of massive damage of the polypeptide chain and the presence of protein molecule parts in the solution. The fraction of individual molecules after exposure to laser radiation reduces the intensity of light scattering, although it does not change the position. In this case, the fractions of aggregates after exposure to laser radiation increase in size and have a high intensity of light scattering; that is, aggregation actively occurs in BSA solutions.

## 4. Materials and Methods

### 4.1. Laser Exposure

**Figure 10:**
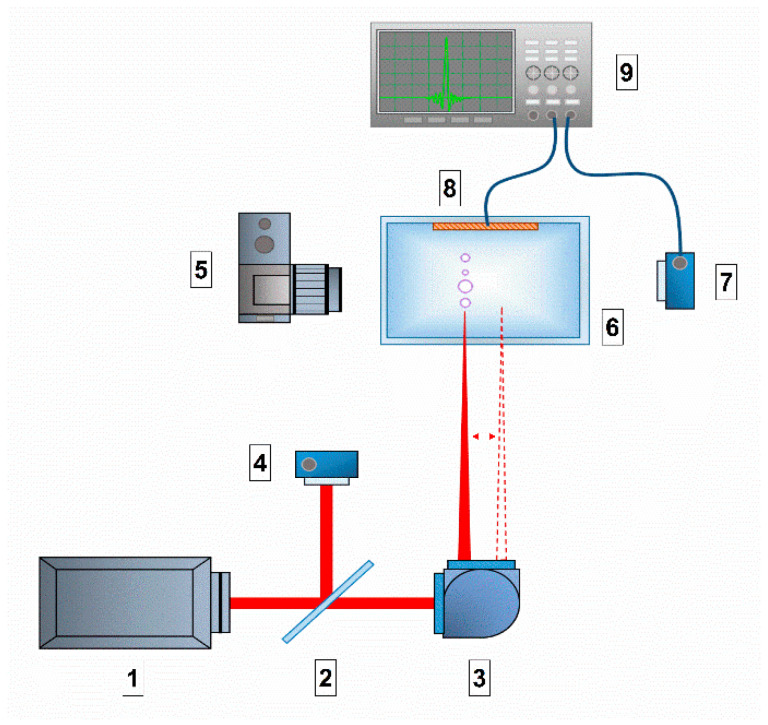
Schematic representation of the experimental setup: 1. laser source; 2. mirror; 3. Galvano-mechanical system of mirrors; 4. pin photodiode; 5. digital camera; 6. glass cell with solution; 7. pin photodiode; 8. acoustic membrane; 9. digital oscilloscope.

A schematic representation of the experimental setup is shown in Figure 10. An Nd:YAG laser NL300 (Ekspla, Vilnius, Lithuania) with the following parameters was used as a laser radiation source: pulse duration τ = 4 ns, frequency υ = 1 kHz, wavelength λ = 532 nm, pulse energy ε = 2 mJ. The laser radiation was focused on the cell center of the cell and moved along a straight line 1 cm long at a speed of 500 m/s using a galvano-mechanical system of mirrors. The diameter of the laser beam at focusing was approximately 30 µm. The movement of radiation in the cell was due to the need to initiate a breakdown in an unperturbed medium and also to avoid thermal defocusing and additional scattering on the bubbles of the resulting gas. Part of the laser radiation was redirected by a mirror (reflection coefficient 5%) to a pin photodiode in order to trigger the time sweep of the oscilloscope. The prepared protein solution was placed in a 25 mL glass cuvette. Inside the cuvette, on one of the walls, a piezo-film acoustic sensor was attached, parallel to the scanning line. A pin photodiode was installed at a distance of 3–4 cm from the cuvette to detect plasma flashes. The signals from the sensor and photodiode were recorded using a GDS-72204E digital oscilloscope (GW Instek, Xinbei, Taiwan). The plasma flashes were photographed using an EOS 450D (Canon, Tokyo, Japan) digital camera (exposure time 10 ms, ISO 800). For each experimental point, there were at least 50 photographs in one series. Acoustic signals from the sensor and plasma signals from the pin photodiode were analyzed using specially developed LaserCav software (https://drive.google.com/drive/folders/1WQmaSCA4mx2HyRSCxtSiik5MWNku9piR (26 September 2022)). The plasma images from the camera were analyzed using the LaserImage program (https://drive.google.com/drive/folders/1YRNF2p7qpejlGP55QBiqM108LSGAseaE (26 September 2022)).

### 4.2. Absorption Spectra

Absorption spectra were measured on a Cintra 4040 (GBC Scientific Equipment, Braeside, Australia) in quartz cuvettes with an optical path length of 10 mm at room temperature (~22 °C). The BSA concentration was 0.5 g/L. The absorption spectra were measured with six-eight samples for each group.

### 4.3. Raman Spectroscopy

The Raman spectra were recorded on a Senterra II Raman Microscope (Bruker Optik GmbH, Karlsruhe, Germany). The spectra were taken from droplets of aqueous solutions of BSA dried on a CaF_2_ substrate at a concentration of 5 g/L in the control and after 30 min laser exposure. The device parameters were as follows: radiation with a wavelength of 532 nm focused with a 50 objective. The laser power was 12.5 mW, and the accumulation time was 2 s. Averaging over 100 spectra was performed. Each sample was measured at a minimum of 3 points to ensure spectral reproducibility. The obtained spectra were processed by applying the following: (1) concave rubber band correction, (2) Min-Max normalization and (3) smoothing (number of smoothing points = 17) in the OPUS 8.2.28 program (Bruker Optik GmbH, Karlsruhe, Germany) [49].

### 4.4. Dynamic Light Scattering

Zetasizer ULTRA Red Label (Malvern Panalytical Ltd., Malvern, UK) was used to obtain information on hydrodynamic particle diameters. A 1 mL solution of BSA with a concentration of 0.4 mg/mL was measured in a plastic cuvette at 25 °C. Five independent experiments were carried out for the control and for each point of influence. The intensity distributions of the hydrodynamic diameters were calculated using the ZS Xplorer program and algorithm [50].

### 4.5. Fluorescence Spectroscopy

The fluorescence of BSA in water was recorded on a Jasco FP-8300 spectrometer (JASCO Applied Sciences, Dartmouth, NS, Canada). Measurements of a 1.8 mL solution of BSA with a protein concentration of 5 g/L were carried out in quartz cuvettes with an optical path length of 10 mm at room temperature (~22 °C). Each sample was measured three times. The Figures show typical spectra: with repeated measurements, the intensity maxima changes by several percent [51].

### 4.6. Viscosity Measurement

A SmartPave 102 rheometer (Anton Paar GmbH, Graz, Austria) was used to obtain the viscosity data of protein solutions. The measuring set was DG26.7 with C-PTD200 cell with 3.8 mL of each sample. All measurements were made at the temperature 25 °C, reducing shear rate from 1000 to 100 s^−1^, using using RheoCompass ™ software (Anton Paar GmbH, Austria). The concentration of proteins in solution was 5 g/L. The main procedures used in determining the viscosity are described above [52].

### 4.7. Refractometry

Refractive index measurements were carried out on a Multiwavelengths Refractometer: Abbemat MW (Anton Paar, Graz, Austria). In the experiments, 1 mL of the solution was poured into the cell of the device, and measurements were made at a wavelength of 435.8, 589.3 and 632.8 nm at a temperature of 25 °C.

## 5. Conclusions

Thus, it has been shown that optical breakdown in protein solutions proceeds with high efficiency, and the formation of plasma and acoustic oscillations is observed. At the same time, the physicochemical characteristics of optical breakdown depend rather weakly on the concentration of protein molecules, which distinguishes the BSA solution from other aqueous solutions containing metallic nanosized objects. The regularities observed when an aqueous solution of BSA is irradiated with laser for different time periods are very similar to the regularities observed in aqueous colloidal solutions of other nanoscale objects. It has been established that after exposure to laser radiation on an aqueous solution of BSA, a decrease in the optical density of protein solutions is observed, both in the absorption maxima of aromatic amino acid residues and in the longer wavelength region. At the same time, the fluorescence intensity of the BSA solution, caused by aromatic amino acid residues, significantly decreases after exposure to laser radiation. Whereas the positions of the excitation and emission maximums do not change, the shape of the fluorescence spot on 3D maps also does not change significantly. It can be argued that the degradation of aromatic amino acid residues occurs under the action of optical breakdown. No significant changes were observed in the Raman spectrum after exposure to laser radiation, with the only change observed being in the region of 1570 cm^−^^1^. This change is associated with the restructuring and degradation of α-helices. Changes in the structure of α-helices usually lead to the partial denaturation or aggregation of molecules. At the same time, there were no significant differences in the refractive index measured at different wavelengths, that is, the total area of protein molecules did not change. It has been established that optical breakdown leads to an increase in the viscosity of aqueous BSA solutions and also reduces the pseudoplasticity of the colloidal solution. Using the DLS method, it was found that there is no massive damage to the polypeptide chain. On the contrary, when exposed to optical breakdown, intense aggregation is observed, while aggregates with a size of 400 nm or more appear in the solution. Thus, under the action of optical breakdown induced by laser radiation in a BSA solution, the processes of partial denaturation and aggregation prevail, aromatic amino acid residues are damaged to a lesser extent, and essential fragmentation of protein molecules is not observed.

## Data Availability

Data available on request due to restrictions eg privacy or ethical.
